# Eating disorders in musicians: a survey investigating self-reported eating disorders of musicians

**DOI:** 10.1007/s40519-017-0414-9

**Published:** 2017-07-14

**Authors:** Marianna Evangelia Kapsetaki, Charlie Easmon

**Affiliations:** 1 Division of Brain Sciences, Imperial College London, London, UK; 2 Division of Surgery and Interventional Science, University College London, London, UK

**Keywords:** Eating disorders, Musicians, Perfectionism, Stress, Anxiety, Depression

## Abstract

**Purpose:**

This study aimed to estimate the prevalence of eating disorders (EDs) in musicians, and to evaluate their relation to perfectionism, stress, anxiety and depression.

**Methods:**

It examined: (1) the prevalence of EDs using the Eating Disorder Examination Questionnaire (EDE-Q), body mass index (BMI) and self-reports, (2) psychological risk factors using the Depression Anxiety Stress Scale (DASS-21) and perfectionism inventory and (3) demographic details, information about musical and career development, lifestyle, eating habits and health. A survey was distributed worldwide and a total of 301 English-speaking musicians aged 18 years and older participated.

**Results:**

Our screening tools for EDs showed a high prevalence of EDs in musicians: the EDE-Q Global Score (EDE-QGS) showed pathological values in 18.66% of the musicians and when questioned about lifetime prevalence, 32.3% of the musicians answered positively. The median BMI was within the normal range. Regarding general mental health, the DASS-21 showed that depression and stress were severe, anxiety was extremely severe and the perfectionism inventory composite score was 26.53. There was no significant difference on the EDE-QGS between musicians who perform different types of music, but music students, professionals, soloists and musicians travelling overseas had a higher percentage of pathological EDE-QGS. Perfectionism was higher in classical musicians and there was a low positive correlation between EDE-QGS and the risk factors: perfectionism, depression, anxiety, stress, peer pressure and social isolation.

**Conclusion:**

EDs are prevalent in musicians and possible risk factors are their increased perfectionism, depression, anxiety and stress due to the demands of their job.

**Electronic supplementary material:**

The online version of this article (doi:10.1007/s40519-017-0414-9) contains supplementary material, which is available to authorized users.

## Introduction

Of all the mental illnesses, eating disorders (EDs) have the highest mortality rate [31]. Eating disorders are mental disorders characterized by abnormal eating habits and they have a huge impact on an individual’s health and well-being. They include: anorexia nervosa (AN) with its restrictive and bulimic sub-types, bulimia nervosa (BN) with its purging and non-purging sub-types, binge eating disorder (BED), pica, rumination disorder, avoidant/restrictive food intake disorder and feeding or eating disorders not elsewhere classified (FEDNEC). Although EDs are identified mostly in females, males also have a high incidence despite being underdiagnosed [37].

Adequate nutrition and a balanced diet are crucial to having a healthy lifestyle, particularly for musicians whose careers are at stake. Eating Disorders can affect them in many ways, e.g. voice dysfunction due to vomiting or gastroesophageal reflux disease (GERD) as part of BN [6, 10, 34], increased injuries due to muscle atrophy especially in females [8], also due to osteoporosis [43], koilonychia [40], fatigue and impaired brain function [43].

In addition, musicians have an increased likelihood of developing an ED, because EDs are related to various developmental and biopsychosocial factors which are present in many musicians’ lifestyle and training. A musician’s unpredictable work schedule, performing and low income are major factors which both from a mental (loneliness, anxiety, depression, personality disorders, substance abuse) and practical (irregular meals when travelling) aspect draw them into a vicious circle of unhealthy eating [7, 11, 35, 42]. Other possible risk factors for EDs in musicians are the cultural idealization of thinness and attractiveness which is particularly common in the music industry, especially nowadays in the constant eye of the media [17, 39], pressure from parents and teachers, competitiveness, peer pressure especially within groups of musicians, and puberty which constitutes a major transition and weak point in the control of musicians’ eating habits as this is a time when they attempt launching their careers and experience heightened body awareness and increased eating concerns [16, 28, 32, 36].

Musicians may avoid reporting any health issues such as an ED, because of pressure from managers and fear of losing a long-awaited career [22]. Therefore, identifying and highlighting the possible abnormal eating patterns within this profession will enable health professionals to recognize the extra pressures of being a musician and provide special care to optimize their health and in turn their performance.

Although EDs have been extensively researched in dancers [5], there has been to date limited research in musicians. Most studies have been small-scale studies and with contradictory results. Some have shown that music students have no difference in the prevalence of EDs compared to non-music students [13], and have a low eating attitudes test (EAT) score and no clinically identified AN [15]. In contrast, over a third of symphony orchestral musicians and over 80% of opera singers were classified as having orthorexia nervosa, an ED which is not reported as an official diagnosis in the diagnostic and statistical manual of mental disorders (DSM) [3, 4].

## Aims

The purpose of this study was to investigate EDs in musicians, considering that they are prevalent among performing artists [3, 5, 44]. We aimed to assess if the prevalence depends on the following factors: the type of music which is performed, the musicians’ income, the stage in their career, the time of year, their age, gender and EDs risk factors, e.g. parental and peer pressures, social isolation and perfectionism. We compared the prevalence of EDs between singers, instrumentalists, soloists and musicians who are performing within a group and we examined if EDs affected their performance. We assessed if touring affected the musicians’ diet, in what way and whether they use any particular foods (or substances) to enhance their performance. In addition, we examined the comorbidity of EDs with other medical conditions, e.g. depression, anxiety, stress, personality disorders, perfectionism, substance abuse, gastrointestinal disorders, and sought to establish if these differed between age or body mass index (BMI) groups and then explored if there is a causative effect, i.e. whether the reported BMI is caused by an ED or another medical condition.

## Methods

### Participants

A total of 306 musicians took part in this study. Of the 306 musicians, three saved but did not complete the survey, one was excluded due to the specific age requirements and one was excluded due to answering only one question (answered positively on having an ED) leading to the analysis of 301 data sets. Some questions were not answered by all 301 musicians; therefore, percentages were calculated using the total of musicians who had answered the particular question, not all the 301 musicians (see tables for more details). Participants were self-identified musicians of 18 years old and older of both gender, multinational, at all stages in their careers (students, professionals, amateurs, retired, etc.) and they were recruited between 5th March and 1st June 2016. We used a random sample to avoid any selection bias. This cross-sectional study was undertaken at University College London (UCL) and was approved by the UCL Research Ethics Committee.

### Survey

The “Eating disorders in musicians” self-administered survey consisted of 28 questions. The self-constructed questions (Q1-16b) included information about the participants’ musical development, lifestyle, eating habits, health and demographics. To find the lifetime prevalence of EDs in musicians, we asked if they have ever suffered from any ED including definitions of each one according to DSM-5 [4]. Any current EDs were assessed using the Eating Disorder Examination Questionnaire (EDE-Q 6.0) [14], in which scores in the range of 0–2.99 were defined as non-pathological and scores in the range of 2.99–6 were defined as pathological [19]. General mental health was assessed using the Depression Anxiety Stress Scale (DASS-21) [30] and eight questions from the perfectionism inventory (Q16c-j) [20] were also included. The participants’ BMI was calculated based on their self-reported height and weight, and categorized using the BMI groups defined by the World Health Organization [46]. Age groups were defined by decade starting from 18 years old.

All questions were uploaded on UCL Opinio 7.3 (online survey software) as one web-based survey in the English language and the advertisement was sent with the survey link to musicians worldwide together with an informed consent form as a welcome message on the Opinio website. Confidentiality and anonymity were maintained and it is not possible to identify participants from any publications. Participants had the right to withdraw with no penalty. The information was used and reported solely by UCL and was not distributed to any third party. The benefit for participants was that the results and copies of any reports or other publications arising from their participation would be sent by the researchers to all those who forwarded an email address.

### Statistical analyses

Significance levels were set at *p* < 0.05 and all analyses were performed using the Statistical Package for Social Sciences version (SPSS) 23.0 (see supplementary material S3). Most of the data were categorical, so Chi squared test and descriptive analyses were used. To adjust for confounders, we used multiple linear regression. All results were reported as mean ± SD and confidence interval (CI) unless otherwise stated.

## Results

There were a total of 119 male (39.5%) and 182 (60.5%) female participants aged 18-75 years (mean ± SD = 31.37 ± 12.55 years, and median age = 27 years). Regarding the music they perform or teach, musicians responded with: classical (85.8%), jazz (19.5%), pop (15.9%), rock (14.2%), folk (3.9%), rap (2.3%), metal (1.9%), world music (1.6%) and other (17.8%). Of the participants, 82.8% were instrumentalists, 31.3% were singers, 4.5% were composers, 2.2% were musicologists, 1.5% were conductors and 2.4% other.

The stages they were in their musical careers were: music students (37.3%), music teachers (36%), professional performers (36.3%), amateur performers (35.6%), other (9.6%). Most frequently they perform: solo (46.9%), within small group (55.8%), within large group (32.3%), other (4.7%).

### Identifying EDs

To identify EDs, we used the EDE-Q, the BMI and a lifetime prevalence question “Have you ever suffered from any of the following ED?”.

The self-reported BMI median was 23.14 kg/m^2^ (IQR = 20.08–26.23) (Table 1 and S1) and the lifetime prevalence of EDs was 32.3% (95% CI 26.62–37.98%) (females: 41.67%, males: 18.27%) (Table 2).

**Table 1:** EDs, perfectionism, DASS, BMI and other factors

Factors	*N*	Number of included participants	%
**Possible trigger for EDs**		281	
Yes	65		60.7
Exams	27		41.53^a^
Stress	12		18.46^a^
Concerts	11		16.92^a^
Relationship problems	6		9.23^a^
Work schedule	5		7.69^a^
Competitive environment	4		6.15^a^
No	41		38.3
**Eating habits affect their career or performance**		282	
Yes^b^	108		38.3
Positively	51		18.1
Negatively	57		20.2
No	174		61.7
**If they had a higher income, they would change their diet**		297	
Yes	124		41.8
No	173		58.2
**Are dependent or addicted to any particular food or drinks which they feel help them as musicians**		297	
Yes	61		20.5
Caffeine	24		39.34
Chocolate	13		21.31
Alcohol	11		18.03
Tea	8		13.11
No	236		79.5
**Are currently receiving treatment for a medical or psychiatric condition**		258	
Yes	67		26
Depression	21		31.34
Gastrointestinal conditions	12		17.91
Anxiety	11		16.42
Bipolar disorder	5		7.46
Chronic pain syndrome	4		5.97
No	191		74
**Menstrual periods missed over the past 3–4 months**		175	
Yes	25		14.28
*N* = 1	9		5.14
*N* = 2	3		1.71
*N* = 3	5		2.85
*N* = 4	7		4
Took the pill	31	169	18.34
**The medical or psychiatric condition … their ED**			
Preceded	11		
Coincided	16		
Followed	11		
**BMI group**		291	
Severe thinness	3		1
Moderate thinness	4		1.4
Mild thinness	16		5.5
Normal range	171		58.8
Overweight	71		24.4
Obese class I	13		4.5
Obese class II	5		1.7
Obese class III	8		2.7

Factors	Mean ± SD	Number of included participants	95 % CI
**BMI** (median, IQR) (kg/m^2^)	23.14, 20.08–26.23	291	23.42–25.02
Height	1.70 ± 0.9		1.69–1.71
Weight	70.87 ± 20.13		68.55–73.19
**Perfectionism inventory**		289	
Composite	26.53 ± 5.74		25.86–27.19
Concern over mistakes	2.89 ± 1.28		2.74–3.03
High standards for others	3.19 ± 1.2		3.05–3.33
Need for approval	3 ± 1.18		2.86–3.13
Organization	3.79 ± 1.51		3.66–3.93
Perceived parental pressure	2.5 ± 1.3		2.35–2.65
Planfulness	4.08 ± 0.98		3.96–4.19
Rumination	3.23 ± 1.14		3.1–3.36
Striving for excellence	3.79 ± 1.12		3.66–3.92
**DASS-21**		282–287	
Stress	29.82 ± 10.15		28.63–31
Anxiety	23.02 ± 8.66		22.01–24.02
Depression	25.95 ± 10.82		24.68–27.22
**Peer pressure**	2.44 ± 1.08	385	2.32–2.57
**Social isolation**	2.63 ± 1.34	389	2.48–2.79

^a^These percentages are from the participants that answered positively about having a trigger for their ED

^b^Many musicians reported the opposite, i.e. that their career affects their eating habits

**Table 2:** Frequency (and percentages) of EDs in musicians by gender

	Pica	Rumination disorder	Avoidant/restrictive food intake disorder	AN	BN	BED	Night eating syndrome	Total^a^
Female	1 (0.6 %)	3 (1.9 %)	18 (11.5 %)	15 (9.6 %)	16 (10.3 %)	19 (12.2 %)	8 (5.1 %)	80 (41.67%)
95% CI	−0.6 to 1.89%	−0.22 to 4.07%	6.52 to 16.54%	4.99 to 14.23%	5.49 to 15.01%	7.04 to 17.3%	1.66 to 8.58%	33.93 to 49.39%
Male	0	0	4 (3.8 %)	6 (5.8 %)	4 (3.8 %)	9 (8.7%)	3 (2.9 %)	26 (18.27 %)
95% CI			0.15 to 7.53%	1.28 to 10.24%	0.15 to 7.53%	3.25 to 14.05%	−0.32 to 6.09%	10.84 to 25.68%
Total	1 (0.4 %)	3 (1.2 %)	22 (8.5%)	21 (8.1 %)	20 (7.7 %)	28 (10.8 %)	11 (4.2%)	106

Percentages were calculated according to the total N of participants that answered Q10 (females: 156, males: 104)

^a^These values are higher than what is reported in the text because some participants reported more than one ED. There were 12 participants that answered ‘Yes, other’ in the question ‘Have you ever suffered from any of the following EDs?’. The free text answer was evaluated and was categorized accordingly to a Yes or No answer as to whether they had an ED or not. One participant had symptoms/behaviours of anorexia, but not severe or long term enough to be diagnosed and, therefore, was classified as ‘No ED’

The global score of the EDE-Q mean and SD was 1.67 ± 1.43 (17 musicians did not complete the EDE-Q so their EDE-QGS could not be calculated) and the specific items of EDE-Q: restraint (1.49 ± 1.60), eating concern (0.99 ± 1.29) (16 musicians did not answer all the restraint and eating concern questions), shape concern (2.28 ± 1.75) (14 musicians did not answer this category of questions), weight concern (1.95 ± 1.66) (15 musicians did not answer this category of questions). The EDE-QGS was pathological in 18.66% of the musicians in our study. In the subcategory, “restraint” 59 musicians (19.6%) had pathological scores, in “eating concern” 41 musicians (13.6%), in “shape concern” 106 musicians (35.2%) and in “weight concern” 78 musicians (25.9%). A total of 83 musicians (27.6%) should be seen for further examination regarding EDs, after showing higher scores in at least two EDE-Q subcategories. The BMI scores were pathological in 45 individuals of this subgroup (54.2%). The EDE-QGS mean was higher in female (2.02 ± 1.49) compared to male (1.09 ± 1.1) musicians (*p* < 0.001). For female musicians, the mean score for each subcategory was: restraint (1.82 ± 1.68), eating concern (1.27 ± 1.43), shape concern (2.67 ± 1.77), and weight concern (2.32 ± 1.7). For male musicians, the mean score for each subcategory was: restraint (0.939 ± 1.29), eating concern (0.52 ± 0.848), shape concern (1.65 ± 1.53), and weight concern (1.34 ± 1.4).

### Risk factors

Most of our participants spend a great deal of time travelling within one country (84.8%) compared to travelling overseas (14.8%) (6 musicians did not report if they spend a great deal of time travelling overseas or within one country). Most musicians (61.7%) reported that their eating habits do not affect their careers or performance (Table 1). In fact, some reported the opposite, i.e. that their career affects their eating habits. Many musicians (41.8%) would change their diet if they had a higher income and some musicians (20.5%) are dependent or addicted to particular foods or drinks, most commonly caffeine. Of the 43 rock musicians, 32.5% reported that they are dependent or addicted to particular foods or drinks (Tables 1, S1). There was no significant difference on the EDE-QGS between musicians who perform different types of music. More professionals (32.25%) reported having an ED sometime in their lifetime compared to amateur musicians (25.55%) and there was a higher percentage of pathological EDE-QGS in music students vs. music teachers, in professionals vs. amateurs, in soloists vs musicians playing within a small or large group and in musicians travelling overseas vs. travelling within one country (Table S2).

The most commonly reported possible triggers for EDs were: exams, stress and concerts (Table 1). The stage in the career at which musicians’ EDs occurred most frequently was during teenage years (62 out of 79 musicians-78.48%) (46 musicians did not report at which stage in their careers the ED had occurred) and there was a low negative correlation between age and anxiety. There was a moderate positive correlation between stress and perfectionism and a low positive correlation between EDE-QGS and the following risk factors: perfectionism, depression, anxiety, stress, peer pressure, social isolation (Table S1). Perfectionism was higher in classical compared to non-classical musicians by 3.193 points (Table S2) and on a five-point scale (Never-1 to Always-5), the mean score for peer pressure was 2.44 and for social isolation 2.63 (Table 1).

## Discussion

### Statement of principal findings

In this cross-sectional study, we examined EDs in self-identified musicians to gain insight into the prevalence of this mental disorder and the possible risk factors in a musician’s lifestyle that may lead to abnormal eating habits. A survey collected data on EDs prevalence (using the EDE-Q, BMI and self-reports), general mental health demographic details, information about musical and career development, lifestyle, eating habits and health. Consistent with our hypotheses, both our screening tools for EDs (lifetime prevalence and EDE-Q) showed a high prevalence of EDs in musicians. Regarding the lifetime prevalence of EDs, 32.3% of all the musicians questioned answered “yes” and all EDE-Q subcategories showed high scores in both female and male musicians. Music students, professionals, soloists and musicians travelling overseas had a higher percentage of pathological EDE-QGS and there was a low positive correlation between EDE-QGS and the risk factors: perfectionism, depression, anxiety, stress, peer pressure and social isolation.

Musicians in our study who reported a pathological EDE-QGS were 18.66% compared to 8% in musical theatre students in another study and more musicians reported a pathological score in all the EDE-Q subcategories compared to musical theatre students [44]. Further examination regarding EDs should be sought by 29.12% of musicians compared to 10% of musical theatre students. The BMI scores were outside the normal range in 54.2% of these musicians, but none of the musical theatre subgroup. Another study using the ΕΑΤ found that the score of music students from a high expectation setting was 13.7 compared to 21.8 in modelling students, 25.6 in dance students and 15.4 in normal controls [15].

Our participants’ BMI was in the normal range which has also been found in music students [13, 15], and musical theatre and drama students [23, 44]. However, 37% of ‘drum and bugle corps’ had BMI above the normal range [29].

Although we found that there is a higher lifetime prevalence of EDs and a higher percentage of pathological EDE-QGS amongst professional compared to amateur musicians, others have found that a competitive environment alone does not necessarily lead to greater expression of AN [15]. There was no significant difference on the EDE-QGS between musicians who perform different types of music. However, there was a higher percentage of pathological EDE-QGS in soloists vs. musicians playing within a small or large group and in musicians travelling overseas vs. travelling within one country which emphasizes findings of previous studies reporting the added stress of being a soloist and travelling overseas [35]. The stage in the career at which musicians’ EDs occurred most frequently was during teenage years, which is consistent with findings of studies conducted on the general population [41].

Most musicians in our study (61.7%) reported that their eating habits do not affect their careers or performance, compared to 75% of the touring musicians in another study, who reported that certain foods did affect their performance [11]. In our study, many musicians reported that their career affected their eating habits, which is also emphasized in the study mentioned above and is consistent with Parry’s report on the effects of musicians’ hectic schedules on their eating habits [35]. Twenty percent of the musicians uses certain foods or drinks to enhance their performance compared to 7% in dance, drama and musical theatre students [45], and specifically 3.7% of the musicians in our study reported that they are dependent or addicted to alcohol compared to 6.1% of touring musicians who reported “very often or always consuming more than 2 alcoholic drinks daily” [11].

Musicians reported severe levels of stress and depression, extremely severe levels of anxiety, increased levels of perfectionism and their mean score for peer pressure was 2.44 and for social isolation 2.63 (on a five-point scale; never-1 to always-5), which is consistent with the description of how stressful a musician’s life is [35]. There was a moderate positive correlation between stress and perfectionism, an association which has previously been reported in adolescents [1] and college students [9]. Another factor that can induce further pressure to a musician's already stressful life is their relatively low income [35], which also may affect their dietary choices as demonstrated in our study, i.e. 41.8% of the musicians would change their diet if they had a higher income.

## Limitations

Although this study has many strengths, several limitations must be acknowledged.

We could not study musicians under 18 years old who probably have a higher percentage of EDs, particularly the adolescents [41]. There may have been a selection bias due to the title of the research in the advertisement, i.e. more musicians with EDs may have participated out of interest and, therefore, we may have found a higher percentage of musicians with EDs compared to the true frequency (false positive result). Though it could be argued that this did not occur as some EDs patients have anosognosia for their ED [27].

Only musicians who had access to the internet were able to complete the online survey which restricted entries. Also, snowball sampling may have occurred although avoided as much as possible, by sending the survey to many different institutes simultaneously and worldwide. The survey was also just in English, so only English-speaking musicians were able to participate.

Instead of or in addition to the EDE-Q, we could have used other questionnaires, e.g. the EAT. Although the EAT is more popular, the EDE-Q is the gold standard in assessing EDs [14]. Still, it should be emphasized that these tests are screening tools and objective self-report measures and not appropriate as a substitute for accepted diagnostic criteria. Self-reported EDs are also not the most reliable source of evaluating EDs prevalence, although they do provide a simple, easy and quick way of community screening [26]. Also only eight questions (16c-j) were used from the perfectionism inventory, which is not as accurate as using the complete perfectionism inventory.

Participants self-identified as being simply musicians and of either professional or amateur or student status. A clarification regarding their professional status would have given us perhaps more accurate results. Also, there could have been nonmusicians who participated identifying themselves as having strong music identity. We could in this case have added a measure of musician identity such as the musicians’ version of the Athletic Identity Measurement Scale (AIMS) [13]. We could also have asked if participants had any other profession, i.e. being both a dancer and a musician, thus having a higher risk for developing an ED.

### Meaning of the study

The increased EDs in musicians could be due to their increased levels of perfectionism (especially in professional classical musicians), because their main aim is to perform perfectly [12, 24, 33]. A plausible reason why singers have more EDs than instrumentalists is that there is an ambivalent association with their primary instrument, i.e. it is one’s body that makes music. More females may have completed our survey because it appears that more females use social media [2, 21].

Our results have implications for clinicians in the prevention and therapy of EDs in musicians. Clinicians can be made further aware of the increased prevalence of EDs in musicians, which is possibly due to extra pressures, and therefore, provide special care to optimize their health, well-being and in turn their performance.

### Future research

Given that this is one of the first and largest scale studies regarding EDs of adult musicians, there is potential for additional research. Future studies could compare musicians with and without EDs with behavioural tests and neuroimaging to see if there are any differences in brain structure. Qualitative studies could explore why musicians have more EDs, whether the pathological mechanism involves the reward system (musicians want happiness and autonomy) and whether it is because they like to be in control (many orchestral musicians are depressed because they are not in control and part-time jobs of musicians are usually related to having control) [18, 25, 38]. Also, studies could examine the appearance pressures (and their internalization), the attractiveness bias and their relation to EDs in the music industry [39]. For more reliable results, future studies could follow a thorough clinical investigation in diagnosing EDs in musicians.

## Electronic supplementary material

Below is the link to the electronic supplementary material.

Supplementary material 1 (DOCX 42 kb)

## References

[1] Abd-El-Fattah SM, Fakhroo HA (2012). The relationship among paternal psychological control and adolescents’ perfectionism and self-esteem: a partial least squares path analysis. Psychology.

[2] Acquisti A, Gross R (2006) Imagined communities: awareness, information sharing, and privacy on the Facebook. Lecture notes in computer science. Springer, New York, pp 36–58. doi:10.1007/11957454_3

[3] Aksoydan E, Camci N (2009). Prevalence of orthorexia nervosa among Turkish performance artists. Eat Weight Disord.

[4] APA (2013). Diagnostic and statistical manual of mental disorders.

[5] Arcelus J, Witcomb GL, Mitchell A (2014). Prevalence of eating disorders amongst dancers: a systemic review and meta-analysis. Eur Eat Disord Rev.

[6] Balata P, Colares V, Petribu K, de Carvalho Leal M (2008). Bulimia nervosa as a risk factor for voice disorders—literature review. Braz J Otorhinolaryngol.

[7] Braun DL, Sunday SR, Halmi KA (1994). Psychiatric comorbidity in patients with eating disorders. Psychol Med.

[8] Brown SL (2004). Being a female concert pianist: the problems of the body.

[9] Chang EC, Watkins A, Banks KH, Watkins A (2004). How adaptive and maladaptive perfectionism relate to positive and negative psychological functioning: testing a stress-mediation model in black and white female college students. J Counsel Psychol.

[10] Cielo CA, Padilha J, Lima DM (2011). Refluxo Laringofaringeo e bulimia nervosa: alteracoes vocais e larinegas. Revista CEFAC.

[11] Cizek E, Kelly P, Kress K, Mattfeldt-Beman M (2016). Factors affecting healthful eating among touring popular musicians and singers. Med Probl Perform Artists.

[12] Dews CLB, Williams MS (1989). Student musicians’ personality styles, stresses, and coping patterns. Psychol Music.

[13] DiPasquale LD (2012). Harmony or discord: disordered eating and personality traits of college music majors.

[14] Fairburn CG, Beglin SJ (1994). Assessment of eating disorders: interview or self-report questionnaire?. Int J Eat Disord.

[15] Garner DM, Garfinkel PE (1980). Socio-cultural factors in the development of anorexia nervosa. Psychol Med.

[16] Gonzalez A, Clarke SD, Kohn MR (2007). Eating disorders in adolescents. Aust Fam Phys.

[17] Griffiths NK (2009). “Posh music should equal posh dress”: an investigation into the concert dress and physical appearance of female soloists. Psychol Music.

[18] Hackman JR, Lawler EE (1971). Employee reactions to job characteristics. J Appl Psychol.

[19] Hilbert A, Tuschen-Caffier B, Karwautz A, Niederhofer H, Munsch S (2007). Eating disorder examination-questionnaire. Diagnostica.

[20] Hill RW, Huelsman TJ, Furr RM, Kibler J, Vicente BB, Kennedy C (2004). A new measure of perfectionism: the perfectionism inventory. J Pers Assess.

[21] Joinson AN (2008) “Looking at”, “looking up” or “Keeping up with” People? motives and uses of Facebook. In: Proceeding of the twenty-sixth annual CHI conference on human factors in computing systems—CHI’08, pp 1027–1036. doi:10.1145/1357054.1357213

[22] Jones ML (2012). The music industries: from conception to consumption.

[23] Joseph A, Wood IK, Goldberg SC (1982). Determining Populations at risk for developing anorexia-nervosa based on selection of college major. Psychiatry Res.

[24] Kenny DT, Davis P, Oates J (2004). Music performance anxiety and occupational stress amongst opera chorus artists and their relationship with state and trait anxiety and perfectionism. J Anxiety Disord.

[25] Kenny D, Driscoll T, Ackermann B (2014). Psychological well-being in professional orchestral musicians in Australia: a descriptive population study. Psychol Music.

[26] Keski-Rahkonen A, Sihvola E, Raevuori A, Kaukoranta J, Bulik CM, Hoek HW, Kaprio J (2006). Reliability of self-reported eating disorders: optimizing population screening. Int J Eat Disord.

[27] Konstantakopoulos G, Tchanturia K, Surguladze SA, David AS (2011). Insight in eating disorders: clinical and cognitive correlates. Psychol Med.

[28] Lacaille N, Koestner R, Gaudreau P (2007). On the value of intrinsic rather than traditional achievement goals for performing artists: a short-term prospective study. Int J Music Educ.

[29] Levy JJ, Statham WJ, VanDoren L (2013). BMI changes among marching artists: a longitudinal study. Med Probl Perform Artist.

[30] Lovibond S, Lovibond PF (1995) Manual for the depression anxiety stress scales. 2nd edn. Psychology Foundation of Australia, Sydney. http://trove.nla.gov.au/work/30421447. Accessed 3 June 2016

[31] Lozano R, Naghavi M, Foreman K, Lim S, Shibuya K, Aboyans V, Memish ZA (2012). Global and regional mortality from 235 causes of death for 20 age groups in 1990 and 2010: a systematic analysis for the Global Burden of Disease Study 2010. Lancet (London, England).

[32] Macarthur LJ (2008). The drive to strive: exploring the experiences of elite-level adolescent artistic performers.

[33] Mor S, Day HI, Flett GL, Hewitt PL (1995). Perfectionism, control, and components of performance anxiety in professional artists. Cognit Ther Res.

[34] Moraes-Filho J, Cecconello I, Gama-Rodrigues J, Castro L, Henry MA, Meneghelli UG, Quigley E (2002). Brazilian consensus on gastroesophageal reflux disease: proposals for assessment, classification, and management. Am J Gastroenterol.

[35] Parry CBW (2003). Prevention of musicians’ hand problems. Hand Clin.

[36] Pruett KD, Sataloff RT, Brandfonbrenner A, Lederman R (1991). Psychological aspects of the development of exceptional young performers and prodigies. Textbook of performing arts medicine.

[37] Raevuori A, Keski-Rahkonen A, Hoek HW (2014). A review of eating disorders in males. Curr Opin Psychiatry.

[38] Rickert DL, Barrett MS, Ackermann BJ (2013). Injury and the orchestral environment: Part I. Med Probl Perform Art.

[39] Ryan C, Wapnick J, Lacaille N, Darrow A-A (2006). The effects of various physical characteristics of high-level performers on adjudicators’ performance ratings. Psychol Music.

[40] Strumia R (2005). Dermatologic signs in patients with eating disorders. Am J Clin Dermatol.

[41] Swanson SA, Crow SJ, Le Grange D, Swendsen J, Merikangas KR (2011). Prevalence and correlates of eating disorders in adolescents. Results from the national comorbidity survey replication adolescent supplement. Arch Gen Psychiatry.

[42] Tepas DI (1990). Do eating and drinking habits interact with work schedule variables?. Work Stress.

[43] Treasure J, Claudino AM, Zucker N (2010). Eating disorders. Lancet.

[44] Vitzthum K, Endres E, Koch F, Groneberg DA, Quarcoo D, Wanke E, Mache S (2013). Eating behavior and nutrition knowledge among musical theatre students. Med Probl Perform Artist.

[45] Werner MJ, Rosenthal SL, Biro FM (1991). Medical needs of performing arts students. J Adolesc Health.

[46] WHO (2016) WHO: global database on body mass index. Retrieved June 9, 2016. http://apps.who.int/bmi/index.jsp?introPage=intro_3.html

