# Supplementary material for: Eating disorders in musicians: a survey investigating self-reported eating disorders of musicians
Source: Eat Weight Disord. 2017 Jul 14;24(3):541–9. doi: 10.1007/s40519-017-0414-9 (PMC6531399; doi:10.1007/s40519-017-0414-9)
Supplement: Supplementary file 1 — Supplementary material 1 (DOCX 42 kb) [file 40519_2017_414_MOESM1_ESM.docx]

**Supplementary material**

**Self-constructed questions no.1-16 of online survey**

1. **Gender**

Male 🞎 Female🞎

1. **Age:**
2. **Weight:……**kg
3. **Height:……….** metres
4. **What type of music do you perform/teach?**

Pop🞎 Rock🞎 Jazz🞎 Classical🞎 Rap🞎 Other🞎 (please specify)

1. **Are you a:**

Singer🞎 Instrumentalist🞎 other🞎(please specify)

1. **What stage are you at in your musical career?**

Music Student🞎 Music Teacher🞎 Professional performer🞎 Amateur performer🞎 Other🞎 (please specify)

1. **If you are a performer, most frequently you perform as (tick one or more):**

Solo🞎 Within small group🞎 Within large group🞎 Other🞎 (please specify)

1. **Do you spend a great deal of time travelling overseas  or are you the majority of the time travelling within one country?**

Overseas 🞎 One country 🞎

1. **Have you ever suffered from any of the following EDs?**

- 🞎**No**
- 🞎**Yes, Pica**= Persistent eating of non-nutritive, non-food substances (e.g. [paper](https://en.wikipedia.org/wiki/Paper), [clay](https://en.wikipedia.org/wiki/Clay), paint, [metal](https://en.wikipedia.org/wiki/Metal), [chalk](https://en.wikipedia.org/wiki/Chalk), [soil](https://en.wikipedia.org/wiki/Soil), [glass](https://en.wikipedia.org/wiki/Glass), [sand](https://en.wikipedia.org/wiki/Sand)) over a period of at least 1 month.
- 🞎**Yes, Rumination Disorder**= Repeated regurgitation of food over a period of at least 1 month. Regurgitated food may be re-chewed, re-swallowed, or spit out.
- 🞎**Yes,** **Avoidant/Restrictive Food Intake Disorder=** eating disturbance, failure to meet nutritional needs with at least one of:
  1. Significant weight loss
  2. Significant nutritional deficiency
  3. Dependence on enteral feeding/oral nutritional supplements
- 🞎 **Yes,** **Anorexia Nervosa=**Restriction of energy intake relative to requirements, leading to a significantly low body weight
- 🞎 **Yes,** **Bulimia Nervosa=** Recurrent episodes of binge eating (large amount of food and loss of control over eating) with compensatory behaviours (self-induced vomiting; misuse of laxatives, diuretics; fasting; or excessive exercise)
- 🞎 **Yes,** **Binge-Eating Disorder=** Recurrent episodes of binge eating without inappropriate compensatory behaviours (e.g. vomiting)
- 🞎 **Yes,** **Night eating syndrome**= Recurrent episodes of night eating, as manifested by eating after awakening from sleep or by excessive food consumption after the evening meal.
- 🞎 **Yes,** **Other**(please specify)

1. **a) In what period in your career (studying, travelling etc.)?**

.............................................................................

**b) Was there a possible trigger (e.g. concerts/exams)?**

🞎Yes (please describe)……………………. 🞎No

1. **Do your eating habits affect your career/performance?**

**Yes, positively**🞎 **Yes, negatively**🞎 **No**🞎

- 1. **Please specify how ...............................................**

1. **Would your diet change if you had a higher income or are you content with what you eat?**

Would change🞎 Would not change🞎

1. **Are you dependent/ addicted to any particular foods/drinks which you feel helps you as a musician (memory/anxiety etc.)?**

🞎Yes, (please specify) 🞎No

1. **Are you currently receiving treatment for any medical or psychiatric condition (e.g. personality disorders/traits, gastrointestinal)?**

🞎Yes (please specify)……………………………… 🞎No

- 1. **If yes, did this/these condition/s precede, follow or coincide with the diagnosis of your ED**? Preceded🞎 Followed🞎 Coincided🞎

N/A (I have not got an ED)🞎

🞎Other (please specify)…………………….

1. **On a scale of 1-5 (write in box) how do these statements apply to you (Never-1 to Always-5)**
   1. Peer pressure🞎
   2. Social isolation🞎
   3. To me, a mistake equals failure. 🞎
   4. I get upset when other people do not maintain the same standards I do🞎
   5. I spend a great deal of time worrying about other people’s opinion of me🞎
   6. I like to always be organized and disciplined 🞎
   7. My parent(s) put a lot of pressure on me to succeed. 🞎
   8. I find myself planning many of my decisions/actions🞎
   9. If I do something less than perfectly, I have a hard time getting over it. 🞎
   10. I drive myself rigorously to achieve high standards. 🞎

Please write your email ……………………………………….if you would like to receive the final report of this project.

Please note that mental health services are available to contact. There are specific EDs support organisations should you want to seek help such as:

- National Centre for EDs http://eating-disorders.org.uk/
- Eating Disorders Support Charity http://www.eatingdisorderssupport.co.uk/help/helpline
- Eating Disorder Support Service (SEED) http://www.seedeatingdisorders.org.uk/
- Beating EDs Trust (BEAT) https://www.b-eat.co.uk/
- Anorexia and Bulimia Care Organization http://www.anorexiabulimiacare.org.uk/
- National Association of Anorexia Nervosa and associated Disorders (ANAD) <http://www.anad.org/>

Table S1. Correlations and group differences

|  | **r_s_ (Spearman’s correlation)*** | **p-value** |  |
| --- | --- | --- | --- |
| **DASS – age**  **Stress**  **Anxiety**  **Depression** | -0.112 (very low)  -0.264 (low)  -0.180 (very low) | 0.059  <0.001  0.002 |  |
| **DASS- Perfectionism inventory**  **Stress**  **Anxiety**  **Depression** | 0.439 (moderate)  0.352 (low)  0.313 (low) | <0.001 |  |
| **ΕDE-QGS - Perfectionism Inventory** | 0.33 (low) | <0.001 |  |
| **EDE-QGS - DASS**  **Stress**  **Anxiety**  **Depression** | 0.377 (low)  0.306 (low)  0.355 (low) | <0.001 |  |
| **EDE-QGS - age** | 0.009 (very low) | 0.88 |  |
| **EDE-QGS - peer pressure** | 0.237 (low) | <0.001 |  |
| **EDE-QGS - social isolation** | 0.283 (low) | <0.001 |  |
| **Perfectionism Inventory composite - age** | -0.037 (very low) | 0.535 |  |
| **BMI-age** | 0.326 (low) | <0.001 |  |
| **BMI- EDE-QGS** | 0.276 (low) | <0.001 |  |
|  | **points** | **p-value** |  |
| **Perfectionism Inventory composite:**  **Classical > non-classical**  **Pop < non-pop**  **Rock < non-rock**  **Jazz < non-jazz**  **Rap > non-rap**  **Female musicians > male musicians**  **Self-reported EDs**  **Yes > No** | 3.193  0.955  1.590  1.627  0.193  0.57  2.33** | 0.01  0.293  0.097  0.057  0.93  0.413  0.002 |  |
| **EDE-QGS**  **Female musicians > male musicians**  **Classical > non-classical**  **If we take into account the Perfectionism Inventory : Classical < non-classical**  **If we take into account DASS: Classical > non-classical**    **Pop > non-pop**  **Rock > non-rock**  **Jazz > non-jazz**  **Rap > non-rap** | 0.928  0.291  0.017  0.123  0.078  0.264  0.167  0.768 | <0.001  0.203  0.783  0.281  0.435  0.161 |  |
|  | **Proportion (%)** | **p-value** | **N of included participants** |
| **ED lifetime prevalence**  **Rap**  **Rock**  **Jazz**  **Pop**  **Classical**  **Singers**  **Instrumentalists**  **Solo**  **Within small group**  **Within large group**  **Travel overseas**  **Travel within one country**  **Professional**  **Amateur**  **Music teacher**  **Music student**  **Only ED**  **ED and another medical/psychiatric condition** | 50  44.44  38.46  37.2  30.94  41.46  34.11  37.3  33.33  27.5  28.94  32.27  32.25  25.55  39.17  33.67  61.84  38.15 | 0.756  0.222  0.524  0.578  0.469  0.095  0.279  0.129  0.867  0.219  0.892  0.892  0.797  0.126  0.112  0.942 | 6  36  52  43  223  82  214  126  147  80  38  220  93  90  97  98 |
| **Are dependent/ addicted to particular foods/drinks**  **Rock**  **Rap**  **Pop**  **Jazz**  **Classical** | 32.5  28.5  25  17.2  19.9 | 0.042  0.635  0.436  0.588  0.534 | 43  7  48  58  256 |
| *Correlation does not mean that there is a causative effect too  ** This relationship was confounded by the individual DASS-21 scores | | |  |

Table S2. EDE-Q Scores

|  | **N of musicians (%)** | **95% CI** | |
| --- | --- | --- | --- |
| **Musicians who reported pathological EDE-QGS and have missed periods and do not take the pill^1^** | 10 (6.06) | | 2.42%-9.7% |
| **Pathological EDE-QGS in musicians**  **Students**^2^  **Teachers**  **Professionals**  **Amateurs**  **Soloists** (p=0.003)  **Within small group**  **Within large group**  **Travel overseas**  **Travel within one country**  **Diet would change if higher income**  **Diet would not change if higher income**  **Also had another medical/psychiatric condition**^3^ | 24 (22.64)  22 (20.75)  19 (17.92)  15 (15)  34 (26.35)  29 (18.58)  16 (17.2)  8 (20)  44 (18.25)  22 (18.64)  31 (18.78)  17 (35.41) | | 5.22%-11.68%  13.03%-28.47%  10.62%-25.22%  8.01%-21.99%  18.75%-33.95%  12.48%-24.68%  9.53%-24.87%  7.61%-32.39%  13.38%-23.12%  11.62%-25.66%  12.82%-24.74%  21.88%-48.94% |
| ^1^Among female musicians that answered all three questions (N=165)  ^2^This means that among student musicians, 22.64% had pathological EDE-QGS.  ^3^This means that among musicians who had a pathological EDE-QGS, 35.41% also had another medical/psychiatric condition | | | |

**S3. Detailed Statistical analyses of research aims**

- To find if there any difference regarding gender of the musician in the lifetime prevalence of EDs I did a chi-square test.

H0: The proportion of EDs is equal in female and male musicians

HA: The proportion of EDs is not equal in female and male musicians.

- To find if the EDE-Q, Perfectionism Inventory, DASS-21, peer pressure, social isolation scores differs between age and BMI groups I used One-way ANOVA after making sure that all the assumptions were satisfied.
  - H0: All age groups’ EDE-QGS means are equal

HA: At least one of the age groups’ EDE-QGS mean differs

- - H0: All age groups’ Perfectionism Inventory score means are equal

HA: At least one of the age groups’ Perfectionism Inventory score mean differs

- - H0: All age groups’ individual DASS-21 scores’ means are equal

HA: At least one of the age groups’ individual DASS-21 scores’ mean differs

- - H0: All age groups’ peer pressure score means are equal

HA: At least one of the age groups’ peer pressure score mean differs

- - H0: All age groups’ social isolation score means are equal

HA: At least one of the age groups’ social isolation score mean differs

- - H0: All BMI groups’ EDE-QGS means are equal

HA: At least one of the BMI groups’ EDE-QGS mean differs

- - H0: All BMI groups’ Perfectionism Inventory score means are equal

HA: At least one of the BMI groups’ Perfectionism Inventory score mean differs

- - H0: All BMI groups’ individual DASS-21 scores’ means are equal

HA: At least one of the BMI groups’ individual DASS-21 scores’ mean differs

- - H0: All BMI groups’ peer pressure score means are equal

HA: At least one of the BMI groups’ peer pressure score mean differs

- - H0: All BMI groups’ social isolation score means are equal

HA: At least one of the BMI groups’ social isolation score mean differs

- To find if there is any relation between age of the musician and their EDE-QGS I did Spearman Correlation as the values weren’t normally distributed.

H0: The correlation between age and EDE-QGS is equal to zero.

HA: The correlation between age and EDE-QGS is not equal to zero.

- To find if the frequency of EDs depend on the type of music which is performed I did a chi-square test.
- To find if EDs are more frequent in singers compared to instrumentalists and if they are more frequent in musicians who play solo compared to playing in a group I did a chi-square test.
- To find if EDs are more frequent in musicians who travel overseas compared to within one country I did a chi-square test.
- To find if musicians with EDs also have other medical or psychiatric conditions e.g. depression, anxiety, substance abuse, personality disorders, gastrointestinal I did a chi-square test.
- Which are the risk factors of EDs in musicians? Could we suggest any possible causes? Possible risk factors for EDs in musicians: parental pressure, peer pressure, social isolation (Question 16a, b). For this I did Spearman Correlation as the values weren’t normally distributed.
- Other hypothesis: Perfectionism (common in musicians) might predispose to an ED (Questions 16c-j). For this I did Spearman Correlation as the values weren’t normally distributed.
- To find the lifetime prevalence of EDs in professional, amateur musicians, music teachers and music students, I did a chi-square test. To adjust for age I performed binary logistic regression.
- Are certain types of music associated with higher rates of dependency/ addiction to particular foods / drinks which musicians feel that it helps them? I did a chi-square test.
- To compare the EDE-Q and Perfectionism Inventory results of our musicians with the general population, I performed a Q-Q plot and saw that the distribution of the values of each EDE-Q score and Perfectionism Inventory were not normally distributed. The Boxplot for the EDE-Q scores showed that they weren’t symmetrical and thus I performed a non-parametric test for asymmetric data, the Sign test. The Boxplot for the Perfectionism Inventory scores showed that they were symmetric and thus I performed a non-parametric test for symmetric data, the Wilcoxon Signed Ranks test.
